# Adaptive Proton Therapy of Pediatric Head and Neck Cases Using MRI-Based Synthetic CTs: Initial Experience of the Prospective KiAPT Study

**DOI:** 10.3390/cancers14112616

**Published:** 2022-05-25

**Authors:** Christian Bäumer, Rezarta Frakulli, Jessica Kohl, Sindhu Nagaraja, Theresa Steinmeier, Rasin Worawongsakul, Beate Timmermann

**Affiliations:** 1 West German Proton Therapy Centre Essen, 45147 Essen, Germany; rezarta.frakulli@uk-essen.de (R.F.); kohl.jessica95@gmail.com (J.K.); sindhunagarajas@gmail.com (S.N.); theresa.steinmeier@uk-essen.de (T.S.); rasin_deck@yahoo.co.uk (R.W.); beate.timmermann@uk-essen.de (B.T.); 2 University Hospital Essen, 45147 Essen, Germany; 3 West German Cancer Center (WTZ), 45147 Essen, Germany; 4 German Cancer Consortium (DKTK), 69120 Heidelberg, Germany; 5 Department of Physics, Technische Universität Dortmund, 44227 Dortmund, Germany; 6 Department of Particle Therapy, 45147 Essen, Germany; 7 Radiation Oncology Unit, Department of Diagnostic and Therapeutic Radiology, Ramathibodi Hospital, Mahidol University, Nakhon 73170, Thailand

**Keywords:** proton therapy, pediatric radiation oncology, adaptive radiation therapy, MRI-guided radiation therapy, head-and-neck tumors, synthetic CTs

## Abstract

**Simple Summary:**

Radiation therapy with protons facilitates highly conformal dose distributions. Thus, normal tissue can be spared effectively, which is a benefit, especially for children. Magnetic resonance imaging (MRI) is ideally suited to assess anatomical changes during the radiation course due to its superior soft tissue contrast and non-existent X-ray exposure. The MRI data have to be transformed to X-ray computed tomography (CT) images, which form the basis of treatment planning. This is conducted by capturing anatomical deformations between MRIs acquired at different times and by warping the planning CT according to these deformations. This procedure was applied in a prospective study enrolling pediatric head and neck cases. The preliminary evaluation of eleven patients with mainly rhabdomyosarcoma diagnosis and at craniofacial and base of skull tumor sites show that neither the deterioration of the target volume coverage nor an increased dose to organs-at-risk over the treatment course is a concern.

**Abstract:**

Background and Purpose: Interfractional anatomical changes might affect the outcome of proton therapy (PT). We aimed to prospectively evaluate the role of Magnetic Resonance Imaging (MRI) based adaptive PT for children with tumors of the head and neck and base of skull. Methods: MRI verification images were acquired at half of the treatment course. A synthetic computed tomography (CT) image was created using this MRI and a deformable image registration (DIR) to the reference MRI. The methodology was verified with in-silico phantoms and validated using a clinical case with a shrinking cystic hygroma on the basis of dosimetric quantities of contoured structures. The dose distributions on the verification X-ray CT and on the synthetic CT were compared with a gamma-index test using global 2 mm/2% criteria. Results: Regarding the clinical validation case, the gamma-index pass rate was 98.3%. Eleven patients were included in the clinical study. The most common diagnosis was rhabdomyosarcoma (73%). Craniofacial tumor site was predominant in 64% of patients, followed by base of skull (18%). For one individual case the synthetic CT showed an increase in the median $D_{2}$ and $D_{\max}$ dose on the spinal cord from 20.5 GyRBE to 24.8 GyRBE and 14.7 GyRBE to 25.1 GyRBE, respectively. Otherwise, doses received by OARs remained relatively stable. Similarly, the target volume coverage seen by $D_{95\%}$ and $V_{95\%}$ remained unchanged. Conclusions: The method of transferring anatomical changes from MRIs to a synthetic CTs was successfully implemented and validated with simple, commonly available tools. In the frame of our early results on a small cohort, no clinical relevant deterioration for neither PTV coverage nor an increased dose burden to OARs occurred. However, the study will be continued to identify a pediatric patient cohort, which benefits from adaptive treatment planning.

## 1. Introduction

With the recent advances in the treatment modalities, the overall survival of pediatric cancer patients has improved. Therefore, the focus of the tumor management has shifted from tumor control to also minimizing the side effects of the treatment including the risk of secondary malignancies and preserving the quality of life [1,2].

Twelve percent of all solid tumors in children develop in the head and neck region [3]. Radiation therapy (RT) plays an important role in the multimodality treatment approach of these pediatric tumors due to various critical organs at risk being situated next to any target volume. Because of its high dose conformality and decreased integral dose, proton therapy (PT) has gained increased importance particularly when children are concerned, as they are generally more vulnerable for radiation injury. Even though long-term data on PT with regard to efficacy and toxicity are limited, there are multiple studies displaying an improved sparing of OARS like parotid glands, inner ear, and pharyngeal muscles with PT when compared to modern photon therapy [4,5]. This reduction in doses helped to reduce the risk for endocrinopathies [6], xerostomia [7], and dysphagia [8], and improving the quality of life. As little is known about the corresponding adverse normal tissue effects in children, it is aimed to gather more clinical evidence in future [9].

In view of the high conformality and the steep dose fall-off of PT, reliable daily dose delivery is of great importance. Any change in the anatomy of the patient during the treatment course due to various factors like weight change, oedema, or sinusitis can not only lead to a relevant deviation of the coverage of the target volume but also of the dose burden to the OARs [10,11]. Therefore, the need for adaptive proton therapy (APT) planning needs to be evaluated. Previous studies pointed out the need to account for anatomical changes during the course of RT [10,12,13,14] and particularly PT treatment [15,16,17,18], especially for tumors of the head and neck. There is clearly less corresponding evidence for PT of pediatric patients. A photon therapy study used verification CTs (vCTs) [19], which are associated with X-ray exposure for the children. Thus, they form an additional potential source of secondary malignancies [20]. The advantage of magnetic resonance imaging (MRI) over X-ray CT is its freedom from the burden of radiation exposure, the inherent soft tissue contrast and the option to perform functional imaging. These features render MRI a viable imaging modality for adaptive radiation therapy. The current KiAPT study pursues the approach of Kraus et al., who demonstrated the integration of an MRI-based synthetic CT (sCT) into a dose tracking workflow [21]. Anatomical changes between MRI scans before or during RT were captured by a deformable image registration and applied to the reference CT thereby generating the sCT.

The current study can add its early experiences to Refs. [10,12,13,14,15,16,17,18] by prospectively evaluating adaptive high precision PT in children with head and neck or base of skull tumors. The aim of the study is to explore the potential benefits of APT based on sCTs, which are generated with verification MRIs.

## 2. Materials and Methods

### 2.1. Study Set-Up, Imaging, and Treatment Planning

Children under the age of 18 years with a tumor in the head and neck/ base of skull region and with a written informed consent from the parents or guardians were prospectively enrolled. Patients who have received RT previously and who were treated palliatively were not included in the study. Primary tumors of the central nervous system, retinoblastoma and lymphomas were also excluded. The treatment modality (chemotherapy and/or surgery) before PT depended on the histology and staging of the tumor.

Patients were immobilized with a thermoplastic face mask (proton mask from Klarity, Heath, OH, USA) attached to a BoS Headframe™(Qfix, Avondale, PA, USA), which is attached to a short Patlog table (IBA, Lovain-La-Neuve, Belgium). The virtual simulation started with an X-ray CT using a Brilliance BigBore Scanner (Philips GmbH Health Systems, Hamburg, Germany) and a laser bridge (LAP, Lüneburg, Germany). The 3D image sets were created with a 120 kVp helical scan protocol and iterative reconstruction. Immediately after the X-ray CT an MRI scan was performed with an in-house Vantage Titan™(Canon Medical Systems Corporation, Zoetermeer, The Netherlands), which is a 1.5 T open bore scanner. The scans were conducted with a head coil aiming for an optimal image quality. Although the BoS frame and the patient table were MRI compatible, they were not used in the MRI scans, because they did not fit into the head coil. The non-contrast MRI scans proceeded with a T1-weighted Fast Field Echo sequence, which was the only 3D protocol available for the scanner. The reconstructed voxel sizes ranged between 0.4 mm and 0.5 mm in lateral direction an 1 mm and 2 mm in slice direction.

The treatment planning system (TPS) RayStation (versions 7, 9B, 10B; RaySearch Laboratories, Stockholm, Sweden) was employed [22] for image data management, image registration, contouring, plan design, and the dosimetric evaluation. The following concept of target volumes was pursued: Gross tumor volume (GTV), clinical target volume (CTV), planning target volume (PTV), and OARs were contoured as per standard guidelines, e.g., from the ICRU [23], the CWS guidance of European Soft Tissue Sarcoma Study Group, and the EURO EWING 2012 Protocol [24]. The CTV1 included the tumor bed ( primary tumor/ lymph nodes prechemotherapy and/or surgery ± residual tumor before PT) and adjacent volumes considered to contain potential microscopic disease depending on the histology. The CTV2 generally covered the tumor bed (±the potential microscopic margin) and the CTV3 the residual tumor. In the definitive PT setting at least two different PTVs were created (PTV1 and PTV2) and in the adjuvant one mostly two PTVs (PTV1 and PTV2).

Dose distributions were simulated with the Monte Carlo dose engine. Robust optimizations of the spot fluences were carried out with an isotropic 3 mm isocenter shift, which accounts for the set-up uncertainties, and 3.5% density uncertainty. Reference [25] gives an account of the used robust planning technique and the implications for target volume coverage and OAR sparing.

### 2.2. Treatment, Verification Imaging, and Adaptation

The patients were treated with the ProteusPlus therapy machine (IBA), which is based on an isochronous cyclotron with subsequent energy selection system. The clinical fields were applied by a gantry-mounted treatment head operated in pencil beam scanning (PBS) delivery mode. The patient positioning verification in the gantry rooms was performed with orthogonal flat-panel X-ray imagers.

The treatment duration was between 5–6.5 weeks. A verification MRI at half of the treatment was defined in the study protocol. According to the terminology of Acharya et al. [26] “on-treatment imaging” and “anatomy-adapted adaptive radiotherapy” according to Ref. [14] was performed. Regarding the definition of Heukelom and Fuller [27] this study performed an ex_aequo-APT, which aims to preserve the target coverage and to keep the dose burden of OARs within bounds. Furthermore, it will be evaluated retrospectively, if an extra OAR sparing would be possible (OAR-APT according to Ref. [27]).

A new X-ray CT would be performed if the dose coverage of the CTV was affected, i.e., a reduction 5% of $V_{95}$[CTV] [26]. The treatment plan would then be optimized on the new CT. The target volume coverage and OAR doses were compared between the initial PT plan and the optimized plan on the new CT. The toxicity data according to CTCAE Version 4.0 at 3 months follow-up was analyzed to determine the early clinical results of APT.

### 2.3. Image Processing

The image processing was performed in a research release of RayStation (version 7). Many processing steps were automated with IronPhython scripts, which can be embedded into RayStation. The dosimetric evaluation was performed in a study patient database of a clinical release (version 10B) of RayStation. The deformable image registration (DIR) operations were conducted with the ANAtomically CONstrained Deformation Algorithm (ANACONDA) [28], which is a module within RayStation. It employs image intensities and anatomical information, which is provided by contoured ROIs. Both ROIs and points can be used as controlling structures for driving the deformation. ROIs can also be used to define focus regions for the deformation.

**Figure 1:**
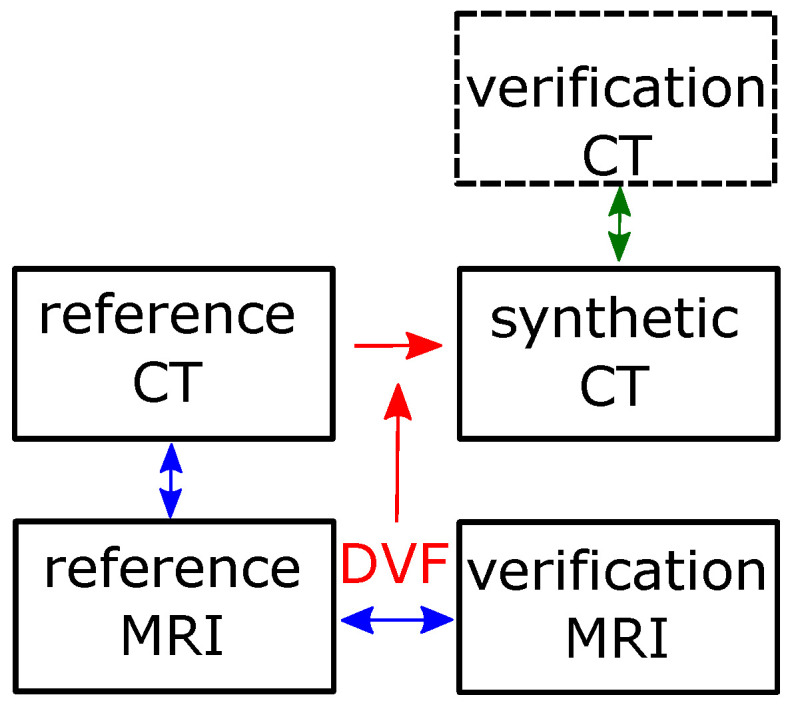
Image processing workflow. Blue arrows indicate the established DIRs. The red arrows indicate the retrieval of the DVF and its use for the generation of the synthetic CT. The green arrows and the block with the dashed frame indicate the validation procedure.

Figure 1 provides an overview of the data workflow. The reference CT is the X-ray CT of the initial scan performed for PT treatment planning. It is sometimes referred to as planning CT. The DIR between reference CT and reference MRI (vertical blue double arrow) provides a data structure, which contains the geometrical relation between the two image data sets. The DIR between reference MRI and verification MRI represents the interfractional anatomical changes. Its data points are designated as DVF. The DVF needs to be adapted to the frame of reference of the reference MRI. This step was performed for convenience outside RayStation in a GNU Octave script. Eventually the sCT is created from the reference CT using the DVF as input with a RayStation function invoked from the embedded scripting environment. The validation of the method is indicated by the vertical green double arrow. The individual tests are described in the next section.

### 2.4. Validation of the Synthetic CTs—Methods

The validation of the sCT method was conducted with the following image data sets:

- A porcine phantom as described in Ref. [29]. The similarity of the vCT and the sCT was evaluated using the target registration error (TRE) method with a <3 mm criterion [29,30].
- A mathematical Shepp–Logan-type phantom. The similarity of the vCT and the sCT was also evaluated with the TRE (<3 mm) and additionally with the Dice similarity coefficient (DSC) (>0.85) criterion [30].
- A clinical case with a full set of planning/verification CT and MRI. A dosimetric comparison was performed.

The porcine phantom had been developed to study the accuracy of DIR [29]. A chunk of porcine meat had been put in a plastic container with movable dividers. The position of the dividers had changed the pressure on the meat, which in turn had induced a deformation. Ten gold fiducials (0.35 mm diameter) had been put into the meat enabling a landmark based evaluation of the registration error. The fiducials were placed all over the phantom including positions with low contrast. In the current study, the X-ray CT (slice thickness of 0.5 mm and pixel size of 0.4 mm × 0.4 mm) and T1-weighted MRI images (slice thickness of 1 mm and pixel size of 1 mm × 1 mm) were used. Two out of four image data sets of Ref. [29] were used for testing during development and the remaining two data sets for the validation. Regarding the evaluation in terms of TRE [30], AAPM TG 138 recommends a tolerance in terms of the maximum voxel dimension, which was assumed to be 2 mm–3 mm. The voxels of the validation image data are clearly smaller, but an impact of the spatial resolution is suspected. A tolerance of 3 mm is assumed, which matches with the root-mean-square TRE estimated from the DIR best algorithm of the original study [29]. The body and bone region of interest (ROI)s were used in the application of the DIR as controlling ROIs and as focus ROIs.

Three-dimensional Shepp–Logan-type phantoms [31] were created. They were adapted to MRI by replacing X-ray CT numbers by typical MRI voxel intensities (Table A1 in the Appendix A) for human tissue. In order to mimic typical variations of tomographic image data, the pixel sizes and slice thickness were arbitrarily set smaller for the MRI data set. Furthermore, a Gaussian filtering was applied to the CT- and MRI Shepp–Logan-type phantoms. In the MRI-type Shepp–Logan phantoms the main axes of the ellipses were increased by 1% to simulate a possible spatial distortion of MRIs. The interfractional anatomical change was simulated by decreasing the volume of one of the ellipses of the Shepp–Logan phantom in the verification image data compared to the reference image data. Depending on the cartesian coordinate the tumor shrinkage was between 13% and 36% (4 mm to 9 mm). This corresponds to the largest anatomical change expected clinically in this study. The body and bone ROIs were used in the application of the DIR as controlling ROI and as a focus ROI. According to Ref. [30] the lower limit for the DSC lies between 0.8 and 0.9, where the lower value is assumed to apply only for small structures.

The clincial test case concerned a 17-year-old male with a relapsing World Health Organization grade II atypical meningioma in the right frontal lobe. The initial treatment plan comprised three fields (right oblique, anterior oblique, vertex) targeting the PTV with a volume of 234.7 cm^3^. A cystic hygroma shrank after the day of the reference CT triggering vCTs together with verification MRIs. In this study, the inital plan was computed on the vCT and the sCT. The quantitative dosimetric comparison was performed by comparing the mean doses to the CTV, the PTV, and the pituitary. The near-maximum dose $D_{1\%}$ was evaluated for the optical nerves and the chiasm. It was checked if the dosimetric values of all these structures are identical for the vCT and the sCT using a student’s t test. A significance level of 5% was required to reject this hypothesis. Furthermore, a gamma-index test was performed with global 2 mm/2% criteria and a 5% dose threshold relative to the prescribed dose. The patient was not part of the KiAPT study but included in the prospective in-house registry KiProReg.

## 3. Results

### 3.1. Validation of the Synthetic CTs—Results

**Figure 2:**
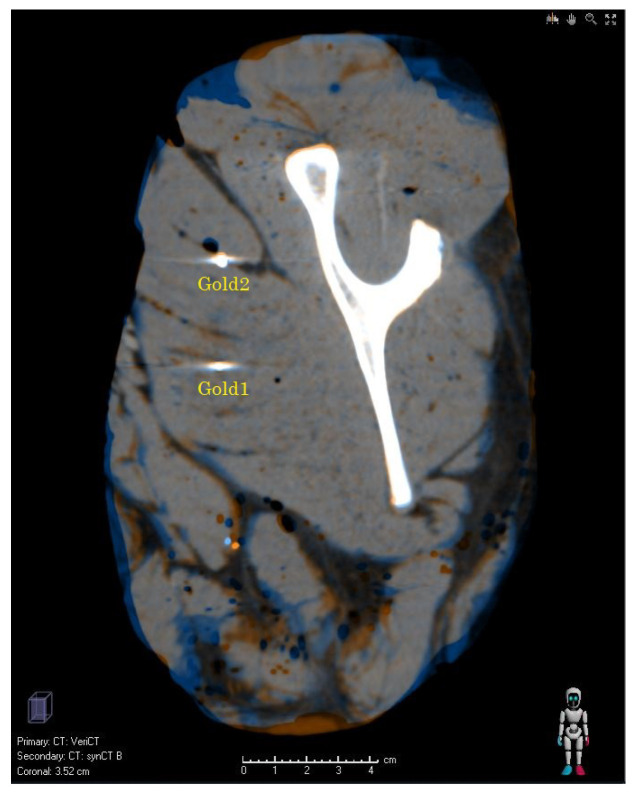
Validation images (coronary view) based on the porcine phantom. The orange (blue) intensity indicates that the CT value of the synthetic CT (verification CT) is bigger.

Figure 2 shows a comparison of the sCT and the vCT in the image domain. By and large, both CT image data sets agree. There are deviations close to the upper edge and the lower edge of the phantom. The agreement of the image data was clearly better in the middle part of the phantom. The distance between the center of the gold marker 1 (2) is 2.4 mm (1.2 mm). The average deviation between gold markers is 3.6 mm (root-mean-square (RSME) 4.3 mm). If the two gold markers, which are within the region of low MRI intensity, are taken out, this value reduces to 2.4 mm (2.5 mm RMSE).

**Figure 3:**
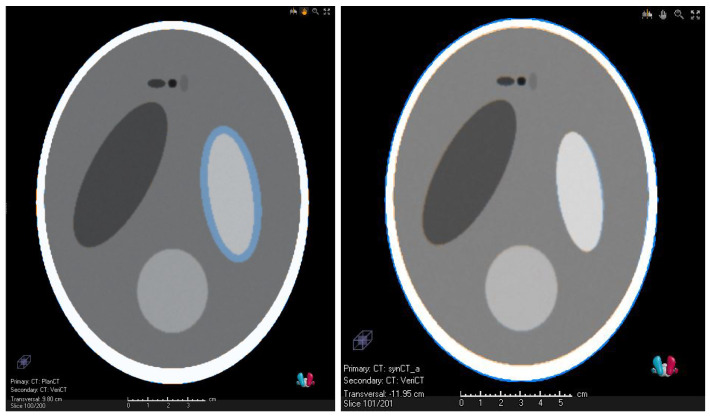
Transversal CT slices concerning the validation with the Shepp–Logan phantom. (**Left**) reference CT and verification CT. They differ with respect to the tumor volume. This is indicated by the blue ring, which indicates higher CT values of the reference CT. (**Right**) Overlay of the synthetic CT and the verification CT.

Figure 3 shows transversal slices of the Shepp–Logan phantom. The reference CT/sCT and the vCT are compared in the left/right image. The contraction of the tumor was modeled by the ellipse on the right hand side, which is indicated by the blue rim in Figure 3 (left). The simulated anatomical change has successfully been reproduced by the deformation-based method as visualized by the comparison of the sCT and vCT (Figure 3 right). Some minor differences, which are mainly located at the right edge of the tumor, are barely visible in Figure 3. All evaluated ROIs of the Shepp–Logan phantom except one had a DSC larger than 0.85. The exception concerned a small ROI with a volume of 0.97 cm^3^ with a DSC > 0.8. The average DSC was 0.95. The TRE ranged between 0.3 mm and 0.9 mm with an average of 0.6 mm.

**Figure 4:**
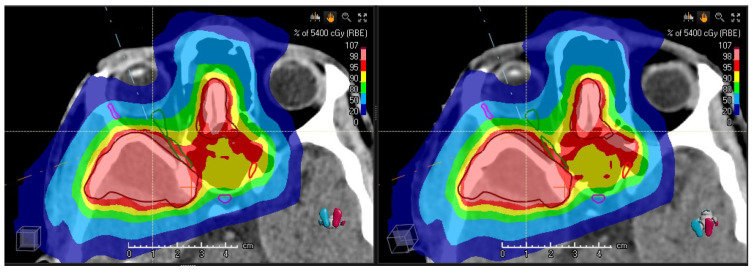
Clinical validation case. **Left**: Dose distribution of the initial treatment plan calculated and overlaid on the verification CT. **Right**: Dose distribution calculated an overlaid on the synthetic CT.

Figure 4 shows exemplary slices together with the dose distributions for the clinical validation case and Table 1 summarizes the quantitative results. The PTV coverage, quantified by the volume fraction, which received at least 95% of the prescribed dose ($V_{95\%}$), was 96.7% for the vCT and 96.8% for the sCT. The maximum dose $D_{1\%}$ was 55.7 GyRBE for the vCT and 55.8 GyRBE for the sCT. The average dose of the PTV was 53.9 GyRBE for both the vCT and the (sCT).

The maximum of the right optical nerve increased from $D_{1\%}=52.5$ GyRBE to 53.3 GyRBE according to the vCT. In contrast, the sCT indicated no change of $D_{1\%}$ (52.5 GyRBE). For the left optical nerve, the vCT and sCT both showed a reduction of $D_{1\%}$ to 52.1 GyRBE, which was initially 52.5 GyRBE in the nominal treatment plan. The maximum dose of the chiasm reduced from $D_{1\%}=52.3$ GyRBE to 51.8 GyRBE (51.7 GyRBE) for the vCT (sCT). The average dose of the pituitary changed from 38.5 GyRBE to 38.6 GyRBE (vCT) and 37.6 GyRBE (sCT). According to a student’s t test comparing the fifth (vCT) and sixth column (sCT) the hypothesis is of a zero dose difference is accepted with a p-value of 0.21. Regarding the comparison of the dose distributions on the vCT and the sCT, the pass rate of the gamma-index test was 98.3%. Voxels with higher gamma-index values were mainly located in the dose fall-off region between the 20% and 5% dose level. The mean (standard deviation) of the gamma-index distribution above the dose threshold was 0.27 (0.21).

### 3.2. Preliminary Results of the Clinical Study

From April 2020 to May 2021, eleven children (six male, five female) fulfilled the inclusion criteria and were enrolled in the study. Table 2 provides an overview of the patient characteristics. The median age of the cohort was 6.9 years (range 1.5–16 years). Rhabdomyosarcoma (RMS) was the most common diagnosis (eight patients), embryonal and alveolar RMS, contributing seven cases and one case, respectively. Other histotypes were Ewings’ sarcoma, chondrosarcoma and mucoepidermoid carcinoma, equally represented with one case. The most common localization was facial with seven cases, followed by base of skull (two cases), orbit (one case) and salivary glands (one cases). The majority of RMS (6) were at paremeningeal site. Chemotherapy was performed as induction or concomitant with PT in eight patients. Nine patients had a radiological residual tumor after induction chemotherapy, following which one of the children which had a R0 surgical resection, and five patients had a R1/R2 surgical resection. PT was administered under sedation in eight cases. The median total prescribed dose was 55.5 GyRBE (50–69.3 GyRBE). Two or three fields were used for each treatment volume. Unilateral neck nodes were treated only in one patient, only one side. No prophylactic lymph nodes irradiation was performed. For RMS patients, RT was administered according to the response to the chemotherapy and the setting (definitive /adjuvant). For patients with residual disease and poor response (<66% reduction after chemotherapy) or in the parameningeal region, three sequential PTVs were delivered with 41.4 GyRBE, 50.4 GyRBE and 55.8 GyRBE (1.8 GyRBE in 33 fractions). In five patients, a simultaneous integrated boost was delivered in order to reduce the patient contact due to the COVID-19 pandemic (42.5 GyRBE, 50 GyRBE and 55 GyRBE in 25 fractions).

The median volume of the GTV before PT and CTV1 was 16.7 cc (range 0.3–45.4 cc) and 63.05 cc (range 27.1–195.8 cc), respectively. The median volume of the parotid gland as OAR was 10.7 cc (range 3.2–28.8 cc) in the left and 10.4 cc (range 4.4–29.2 cc) in the right. The median volume changes, i.e., shrinkages, for the right and left parotid glands were 0.2 cc (range 0.0–1.0 cc) and 0.1 cc (range 0.0–0.3 cc), respectively. The median volume changes for the CTV1 were 0.13 cc (range 0.05–0.20 cc). Table 3 provides an overview of the dose statistics of the OARs. The initial plan, when applied on the sCT, showed an increase in the median $D_{2}$ and $D_{\max}$ dose on the spinal cord from 20.5 GyRBE to 24.8 GyRBE and from 14.7 GyRBE to 25.1 GyRBE, respectively. In one case, the spinal cord dose $D_{2}$ increased from 14.7 GyRBE to 25.1 GyRBE in one patient due to weight loss and tumor response. These changes did not trigger a vCT and a subsequent replanning, since the dose remained well below the tolerance limit of the spinal cord. The change of dose received by other OARs including brainstem, cochlea, optic nerve and chiasm, salivary glands, pharynx and larynx was irrelevant from a clinical point of view. For PTV1, the mean dose to 95% of the volume decreased by 0.1 GyRBE (0.8 GyRBE) on average (maximally) and the volume covered by 95% of the prescribed dose decreased by 0.1% (0.9%) on average (maximally). Early follow-up data were available in eight children. No evidence of tumor after surgery and or chemotherapy and PT was seen in eight patients, partial remission in two patient and stable disease in two patients. No higher grade toxicities (CTCAE V4 > 2) were observed after three months.

## 4. Discussion

### 4.1. Clinical Impact

Precision PT requires tomographic image guidance. As the radiation exposure of the vCTs forms a potential source of secondary malignancies [32], it is essential to incorporate verification MRI scans, especially when anatomical changes in the beam ports necessitate treatment plan adaptation. In the initial phase of the study, the workflow was implemented in a PT center and cases with tumors of the head and neck and base of skull were investigated. Within the selected patients from this cohort, we could not find any significant difference in the OAR doses and target volume coverage in reference CT and sCT. The reason for this could be that the cohort included only pediatric patients with mainly RMS and other kind of pediatric tumor who were pretreated with induction polychemotherapy according to respective protocol. Most of the patients had a favorable response already following induction chemotherapy and further reduction in size of the gross tumor during the course of PT was minimal. In addition, patients of the study did not experience any relevant weight loss during the RT course.

A limitation of the dose evaluation on the sCTs is the lack of a detailed dose perturbation test. While the initial treatment plans have been evaluated according to the robustness approach of the dutch PT centers [33], the analysis of the dose statistics on the sCTs was conducted in a conventional PTV-based manner. According to our experience in clinical planning, the PTV coverage correlates with a CTV coverage of perturbed dose distributions in terms of patient shifts and density offsets for the tumor sites and field configurations of the current study. Because differences in the dose statistics between these evaluation schemes cannot be excluded, the incorporation of robustness should be regarded as a technical improvement for future studies.

### 4.2. Comparison with Previous Studies

In a study by Simone et al., adaptive intensity-modulated PT (IMPT) was compared to non-adaptive IMPT as well as adaptive intensity-modulated RT (IMRT) [34]. Adaptive IMPT was significantly better in terms of OAR sparing, e.g., regarding the average doses to the contralateral parotid gland (p=0.049) and the larynx (p=0.049), but did not show a clinical benefit when compared to non-adaptive PT plan, due to the better dose distribution achieved with PT. This result is also supported by the results of the current study. Volpe et al. [35] also reported quite small dosimetric changes for adjuvant (mixed) PT of children with tumors in the fossa posterior. In comparison, Surucu et al. included patients with squamous cell carcinoma of head and neck which were treated with upfront chemoradiation [13]. They showed a reduction of maximum dose on spinal cord and brainstem, and the mean dose $D_{\mathrm{mean}}$ received by ipsilateral and contralateral parotid glands by 4.5%, 3.0%, 6.2% and 2.5%, respectively, through plan adaptation. Furthermore, Chen et al. showed an inferior 2-year loco-regional control of only 79% without plan adaptation compared to 88% with plan adaptation. All recurrences were observed in the high dose region [12]. Minatogawa et al. compared the results of adaptive IMRT with adaptive IMPT and showed a statistically significant reduction in the $D_{\mathrm{mean}}$ of the right parotid gland, both temporomandibular joints, the oral cavity, the larynx (all with p<0.001) and thyroid gland (p=0.002) when using adaptive IMPT [18]. The retrospective study by Placidi et al. reported about plan adaptations in 5.5% of the studied cases, which were treated with PBS, as a result of anatomical changes during the PT course. Predictive factors and a patient group in which APT is to be recommended were not analyzed [17]. The side effects after three months of an orophangeal patient cohort was evaluated by Sio et al. [16] showing a faster return to normal function with IMPT [15]. The study of Laskar et al. contained 50% of children with nasopharyngeal carcinoma [19]. In contrast to the current study, a significant change in the anatomy, e.g., a mean gross tumor volume reduction of 40% (p=0.005), was observed during the RT course due to the rapid response of these tumors. The current study did not include any nasopharyngeal cancer patients so far. Still, similar to these studies, we observed an increase in the maximum dose and dose received by 2 cc volume of spinal cord and brainstem, when the original plan was applied to the sCT. Hence, optimization of the plan on the sCT or on a vCT, which would be triggered by the evaluation on the sCT, could reduce long term toxicity and improve quality of life. One might also note that the IMPT optimization incorporated perturbed scenarios of the set-up and density, which might also enhance the robustness against anatomical changes. In this regard, the finding of Hague et al. [36] was confirmed. The limited need to replan due to higher OARs doses induced by anatomical changes might be explained by the major advantage of PT: Because the dose of many OARs can be kept low in the initial treatment planning, a moderate increase over the treatment course does not affect the normal tissue complication probability. As pointed out in the review of Morgan and Sher [14], adaptive RT should seek to identify a cohort of patients, which would clearly benefit from replanning, thereby justifying the additional clinical resources. From literature and the clinical course of the disease it is assumed that adaptive PT may be more relevant in nasopharyngeal cancer or craniopharingomas when compared to craniofacial RMS.

A recent review of adult patients with head and neck tumor reported that primary tumor, lymph nodes and parotis start to shrink at the first two weeks of treatment with a tendency to increase the shrinkage rates at four and seven weeks [14]. Median tumor size shrinkage ranged from 3 to 16%, 7 to 48% and 6 to 66% by the end of week 2, 4, and 7, respectively. Involved nodes can also shrink throughout treatment to similar degrees as the primary tumor. The average volume of the parotids has been reported to decrease as much as 14.7, 37, and 48% by the end of weeks 2, 4 and 7 [14]. The study of Volpe et al. [35] performed a verification MRI two weeks after the start of treatment [35]. This agrees with the findings of Ref. [26], which reported changes in the second week (n = 3/11) or third week (8/11). Other previous studies conducted verifications scans after two weeks [37], or after three weeks [38]. Ref. [39] reported changes for 41% occurring by fraction 10/35 and for 55% by fraction 15/35. However, regarding children the data available on tumor or OAR changes in the region of head and neck during radiotherapy are limited so far in the literature [19,40,41]. Exceptions are the data available on craniopharyngioma with a cystic component at the time of the RT, where the size of cyst can increase or decrease during the treatment course [42,43]. Future studies will have to assess the optimal timing for a replanning. The current study scheduled only mid-treatment MRI to avoid excessive burden on the clinical facility. This could be a potential limitation to identify the optimal timing of treatment adaptation. However, in selected cases, e.g., large primary tumors and/or lymph nodes or craniopharyngioma with a cystic component at the time of PT more than one verification MRI would be performed.

### 4.3. Validation of the Method and Technical Considerations and Limitations

Ideally, end-to-end tests would be conducted with a dedicated anthropomorphic phantom with X-ray CT and MRI contrast as, e.g., described in Ref. [44]. Such kind of procedures appears to be restricted to institutes with a strong focus on research. Clinically operating PT centers have limited technical options. Our validation methods (Section 2.4) demonstrated solutions, which require less medical physics resources. The advantage of using a publically available image set is the possible comparison to other studies. The only data set [29] (“porcine phantom”) which was available to the authors, however, suffered from imperfections. For instance, the deviations sCT and vCT observed at the lower and upper edge (Figure 2) were caused by reduced MRI intensities, especially in the bottom part of the phantom. These MRI artefacts affected the quality of the DIR and, thus, propagated into the sCT. On the one hand, these imperfections might reflect realistic circumstances of the clinical routine. On the other hand, they hampered a fully, quantitative evaluation. Thus, there is need to provide validated reference sets similar to Ref. [30]. The used Shepp–Logan phantom might serve as a general benchmark example. The evaluated DSC values with an average of 0.95 are interpreted as passed test according to Ref. [30]. Similarly, the TRE evaluation meets the requirements of AAPM TG 138 [30]. Of course, the Shepp–Logan phantom lacks detailed anatomical structures. This can be overcome by using customized, virtual anatomical phantoms as, e.g., realized in the frame of particle therapy in Ref. [45]. A further limitation of the Shepp–Logan phantom is the simplistic representation of the tomographic imaging. For instance, the complex acquisition and reconstruction methods of MRI were not considered. Reference [46] and references therein provide details about the limitations and show how realistic numerical MRI phantoms can be established.

The used method of sCTs is limited by the DIR software. In the tests with the porcine phantom described in Section 3.1, we observed a slight decreasing trend of the quality of registration with increasing deformation. In the clinical validation case (Figure 4 in Section 3.1) the biggest deviations in terms of dose were 1.5% for right optical nerve and 2.6% for the pituitary gland, which is on the level of 1 GyRBE. This is clearly lower than the 5% criterion defined in Ref. [26] as a trigger level for adaptation of a PT treatment plan. However, the impact of the dose uncertainties on the decision for replanning is not negligible, especially for OARs which are located downstream of at least one proton field as in the validation case (Figure 4). The dose statistics (Section 3.1) of the optical nerves are very demanding tests: The left optical nerve is located at the edge of the high-dose volume, which is also the case for the right optical nerve. Because the latter one also intersects partly with the PTV, a dose level between 95% and 100% of the prescribed dose was pursued for this sub-volume. The chiasm was also located in the distal gradient and the pituitary was located at the field edge. The parametric test of the dose values of the contoured structures (p=0.21) appears to have insufficient power to show the equivalence of vCT- and sCT-based dose computations. Furthermore, this evaluation is restricted to the sub-volumes of the contoured structures. Nevertheless, the high gamma pass rate of 98.3%, which is clearly above 95%, indicates the similarity of the dose distributions. The current study was limited to a single clinical validation case. Ideally, more clinical validation cases with other tumor sites and other kind of anatomical changes should be evaluated. This could be performed in a future extension of the study to tumor entities indicated in Section 4.2.

A limitation concerning the implementation of the clinical study is that immobilization devices could not be used during MRI scans due to hardware restrictions. The current study followed a pragmatic approach by manually reproducing the head inclination of the X-ray CT. This could lead to inaccuracies in positioning, especially in the neck, and subsequently in the registration. As result, the anatomical changes could be underestimated, which in turn could affect the replanning accuracy [47,48]. Recent studies demonstrate the use of immobilization equipment in the MRI [26,48].

MRI comes with the advantage of a better soft tissue contrast compared to X-ray CT. This eases the delineation of RT targets. A native T1 3D sequence without contrast medium was selected in this study since Ref. [49] showed good results of sCTs from a T1-based MRI sequence. Other reasons that we selected only this T1 sequence are a better anatomical visualization in cranial and neck region, a suitable slice thickness to register with planning CT and the short time of anesthesia of young children. Reference [47] discusses T1-weighted sequences for stereotactical applications. Although the distortion of the images is still the major problem for MRI, previous studies showed less than 2 mm of distortion between T1 and T2 sequences with appropriately performed MRI [50,51,52]. Furthermore, there is a clear trend that the distortions get worse with radial distance from isocenter [47] with minor distortions below about 100 mm [51,53]. A similar trend was reported in Ref. [53]. Considering also the young patient cohort and tumor localization of the current study, image distortion was a minor concern as the evaluated contours were within a radius of 100 mm. Of course, care has to be taken regarding sequence-specific distortions, which occur at air-bone interfaces [47]. We did not perform multiple sequences to reduce the motion uncertainty between sequences of MRI.

The current study used sCTs from the DIR between MRIs, which was transferred to the reference CT. This technique was applied for cranial and pelvic cases in Refs. [54,55] and also used to transfer 4D MRI data to the reference CT [45,56,57]. Other studies report about direct sCTs from MRI (see Refs. [58,59,60,61,62] and references therein). Especially, the machine learning based implementation is gaining popularity. A reduction of uncertainty of the so-obtained estimate of the electron density would be desired. In this regard, the facility of the authors recently presented a novel approach integrating time-of-flight PET into MRI-guided PT [63].

The current study employed a research version of a commercial TPS to generate the sCTs. Regarding a translation into clinics, this approach could be regarded as an advantage over previous studies, which used independent research codes for this purpose. It should be stressed that the authors did not use the so-generated sCTs for clinical decisions, thereby adhering to the terms of the vendor of the TPS and the general rules for certified medical devices.

## 5. Conclusions

Adaptive proton therapy, which uses verification MRI scans and deformable image registration to create synthetic CTs, was implemented with simple, realizable, commonly available tools and methods. The achievable potential dose reduction would be relevant for the investigated cohort of vulnerable patients. The evaluation of eleven pediatric patients with mainly rhabdomyosarcoma at craniofacial and base of skull tumor sites indicates that neither the deterioration of the target volume coverage nor an increased dose burden to organs-at-risk over the treatment course is a concern. This demonstrates that proton therapy can be a robust treatment option for selected patient cohorts, thereby sparing the extra clinical resources which are required for a closed-loop adaptive workflow. It would be important to explore which category of cases exhibits a similar anatomical robustness. Thus, the patient cohort under study could be extended by including, e.g., also craniopharyngeoma.

## Figures and Tables

**Table 1:** Clinical validation case. ‘ROI’ refers to the clinical contours. ‘rCT’ refers to the dose evaluation on the reference CT (planning CT), ‘vCT’ on the verification CT, and ‘sCT’ on the synthetic CT.

Quantity	ROI	Unit	rCT	vCT	sCT	Clinical Goal
$V_{95\%}$	PTV	%	98.3	96.7	96.8	95.0
$D_{1\%}$	PTV	GyRBE	55.7	55.7	55.8	57.8
$D_{\mathrm{mean}}$	PTV	GyRBE	54.0	53.9	53.9	≈54
$D_{1\%}$	right optical nerve	GyRBE	52.5	53.3	52.5	56.0
$D_{1\%}$	left optical nerve	GyRBE	52.5	52.1	52.1	56.0
$D_{1\%}$	chiasm	GyRBE	52.3	51.8	51.7	56.0
$D_{\mathrm{mean}}$	pituitary	GyRBE	38.5	38.6	37.6	40

**Table 2:** Patient and tumor characteristics. ‘Pts’: patients, ‘PT’: proton therapy.

Median Age (Range)	6.9 Years (Range, 1.5–16 Years)
**Gender**	
Male	6 pts (55%)
Female	5 pts (45%)
**Site**	
Craniofacial	7 pts (64%)
Base of skull	2 pts (18%)
Other site	2 pts (18%)
**Histology**	
Rabdomyosarcom	8 pts ( 73%)
Other histotype	3 pts ( 27%)
**Treatment before PT**	
Chemotherapy	8 pts ( 73%)
Surgery	6 pts ( 55%)
**N Stage**	
N0	10 pts (91%)
cN+	1 pt (9%)
Median prescribed dose (range)	55.5 GyRBE (50–69.3 GyRBE)

**Table 3:** Therapeutic dose received by various organs-at-risk (OARs) on reference CT and synthetic CT created from the verification MRI.

OAR	Median Dose on Reference CT (Range)	Median Dose on sCT (Range)
	(GyRBE)	(GyRBE)
Spinal cord		
$D_{2}$	20.5 (0.06–48.7)	24.8 (0.06–48.2)
$D_{\max}$	22.7 (0.08–49.7 )	28.1 (0.07–49.2)
Brainstem		
$D_{2}$	31.9 (0.5–54.3)	31.8 (0.5–54.2)
$D_{\max}$	34.8 (0.9–55.5)	34.7 (0.9–55.4)
Optic chiasm		
$D_{2}$	9.9 (0.4–54.7)	9.4 (0.5–54.7)
$D_{\max}$	8.9 (0.4–54.7)	8.3 (0.4–54.6)
Optic nerve (right)		
$D_{2}$	34.6 (1.9–55.4)	34.7 (1.8–55.3)
$D_{\max}$	33.3 (1.9–56.4)	33.3 (1.8–56.5)
Optic nerve (left)		
$D_{2}$	18 (0.1–53.3)	17.9 (0.1–53.5)
$D_{\max}$	18 (0.1–53.4)	17.9 (0.1–53.5)
Parotid gland (mean)		
Right	38.8 (0.2–51.7)	38.7 (0.2–51.8)
Left	0.9 (0–52.1)	1.1 (0–52)
Submandibular gland (mean)		
Right	28.4 (0.04–57.2)	29.1 (0.04–57.1)
Left	5.2 (0–50.4)	5.2 (0–50.4)
Cochlea		
Right (mean)	26.8 (0.9–59.7)	26.6 (0.9–59.8)
Left (mean)	1.3 (0–37.2)	1.03 (0–36)

## Data Availability

Validation data are available on request.

## Abbreviations

The following abbreviations are used in this manuscript:

sCT	synthetic CT
vCT	verification CT
DIR	deformable image registration
TRE	target registration error
IMPT	intensity-modulated PT
IMRT	intensity-modulated RT
ROI	region of interest
MRI	magnetic resonance imaging
DSC	Dice similarity coefficient
DVF	deformation vector field
OAR	organ-at-risk
OARs	organs-at-risk
PBS	pencil beam scanning
PT	proton therapy
RT	Radiation therapy
ART	adaptive photon therapy
APT	adaptive proton therapy
TPS	treatment planning system

## Appendix A. Details of the Shepp–Logan Phantoms

**Table A1:** Voxel intensities assumed in the CT (Hounsfield units) und MRT implementations of the Shepp–Logan phantom.

Soft Tissue	CT	MRT
Air	−1000	0
Skin	−100	5500
Bone	1000	800
Brain, gray matter	0–25	1500–2300
Brain, white matter	25–50	2900–3600
Tumor	50	3600
Craniospinal fluid	−5	1000

## References

[1] Armoogum K.S., Thorp N. (2015). Dosimetric Comparison and Potential for Improved Clinical Outcomes of Paediatric CNS Patients Treated with Protons or IMRT. Cancers.

[2] Leiser D., Calaminus G., Malyapa R., Bojaxhiu B., Albertini F., Kliebsch U., Mikroutsikos L., Morach P., Bolsi A., Walser M. (2016). Tumour control and Quality of Life in children with rhabdomyosarcoma treated with pencil beam scanning proton therapy. Radiother. Oncol..

[3] Albright J.T., Topham A.K., Reilly J.S. (2002). Pediatric Head and Neck Malignancies: US Incidence and Trends Over 2 Decades. Arch. Otolaryngol.–Head Neck Surg..

[4] Fagerström Kristensen I., Nilsson K., Nilsson P. (2015). Comparative Proton and Photon Treatment Planning in Pediatric Patients with Various Diagnoses. Int. J. Part. Ther..

[5] van der Laan H.P., van de Water T.A., van Herpt H.E., Christianen M.E.M.C., Bijl H.P., Korevaar E.W., Rasch C.R., van ‘T Veld A.A., van der Schaaf A., Schilstra C. (2013). The potential of intensity-modulated proton radiotherapy to reduce swallowing dysfunction in the treatment of head and neck cancer: A planning comparative study. Acta Oncol..

[6] Eaton B.R., Esiashvili N., Kim S., Patterson B., Weyman E.A., Thornton L.T., Mazewski C., MacDonald T.J., Ebb D., MacDonald S.M. (2015). Endocrine outcomes with proton and photon radiotherapy for standard risk medulloblastoma. Neuro-Oncology.

[7] Beetz I., Schilstra C., van der Schaaf A., van den Heuvel E.R., Doornaert P., van Luijk P., Vissink A., van der Laan B.F., Leemans C.R., Bijl H.P. (2012). NTCP models for patient-rated xerostomia and sticky saliva after treatment with intensity modulated radiotherapy for head and neck cancer: The role of dosimetric and clinical factors. Radiother. Oncol..

[8] Christianen M.E., Schilstra C., Beetz I., Muijs C.T., Chouvalova O., Burlage F.R., Doornaert P., Koken P.W., Leemans C.R., Rinkel R.N. (2012). Predictive modelling for swallowing dysfunction after primary (chemo)radiation: Results of a prospective observational study. Radiother. Oncol..

[9] Constine L., Ronckers C., Hua C.H., Olch A., Kremer L., Jackson A., Bentzen S. (2019). Pediatric Normal Tissue Effects in the Clinic (PENTEC): An International Collaboration to Analyse Normal Tissue Radiation Dose–Volume Response Relationships for Paediatric Cancer Patients. Clin. Oncol..

[10] Schwartz D.L., Garden A.S., Thomas J., Chen Y., Zhang Y., Lewin J., Chambers M.S., Dong L. (2012). Adaptive Radiotherapy for Head-and-Neck Cancer: Initial Clinical Outcomes From a Prospective Trial. Int. J. Radiat. Oncol. Biol. Phys..

[11] Paganetti H., Botas P., Sharp G.C., Winey B. (2021). Adaptive proton therapy. Phys. Med. Biol..

[12] Chen A.M., Daly M.E., Cui J., Mathai M., Benedict S., Purdy J.A. (2014). Clinical outcomes among patients with head and neck cancer treated by intensity-modulated radiotherapy with and without adaptive replanning. Head Neck.

[13] Surucu M., Shah K.K., Roeske J.C., Choi M., Small Jr W., Emami B. (2017). Adaptive radiotherapy for head and neck cancer: Implications for clinical and dosimetry outcomes. Technol. Cancer Res. Treat..

[14] Morgan H.E., Sher D.J. (2020). Adaptive radiotherapy for head and neck cancer. Cancers Head Neck.

[15] Ramaekers B.L., Pijls-Johannesma M., Joore M.A., Van Den Ende P., Langendijk J.A., Lambin P., Kessels A.G., Grutters J.P. (2011). Systematic review and meta-analysis of radiotherapy in various head and neck cancers: Comparing photons, carbon-ions and protons. Cancer Treat. Rev..

[16] Sio T.T., Lin H.K., Shi Q., Gunn G.B., Cleeland C.S., Lee J.J., Hernandez M., Blanchard P., Thaker N.G., Phan J. (2016). Intensity modulated proton therapy versus intensity modulated photon radiation therapy for oropharyngeal cancer: First comparative results of patient-reported outcomes. Int. J. Radiat. Oncol. Biol. Phys..

[17] Placidi L., Bolsi A., Lomax A.J., Schneider R.A., Malyapa R., Weber D.C., Albertini F. (2017). Effect of Anatomic Changes on Pencil Beam Scanned Proton Dose Distributions for Cranial and Extracranial Tumors. Int. J. Radiat. Oncol. Biol. Phys..

[18] Minatogawa H., Yasuda K., Dekura Y., Takao S., Matsuura T., Yoshimura T., Suzuki R., Yokota I., Fujima N., Onimaru R. (2021). Potential benefits of adaptive intensity-modulated proton therapy in nasopharyngeal carcinomas. J. Appl. Clin. Med. Phys..

[19] Laskar S., Pandit P., Mallik S., Tike P., Chaudhari S., Khanna N., Vora T. (2015). Adaptive radiation therapy for pediatric head and neck malignancies: Dosimetric implications. Pract. Radiat. Oncol..

[20] Chin S., Eccles C.L., McWilliam A., Chuter R., Walker E., Whitehurst P., Berresford J., Van Herk M., Hoskin P.J., Choudhury A. (2020). Magnetic resonance-guided radiation therapy: A review. J. Med. Imaging Radiat. Oncol..

[21] Kraus K.M., Jäkel O., Niebuhr N.I., Pfaffenberger A. (2017). Generation of synthetic CT data using patient specific daily MR image data and image registration. Phys. Med. Biol..

[22] Bäumer C., Geismar D., Koska B., Kramer P., Lambert J., Lemke M., Plaude S., Pschichholz L., Qamhiyeh S., Schiemann A. (2017). Comprehensive clinical commissioning and validation of the RayStation treatment planning system for proton therapy with active scanning and passive treatment techniques. Phys. Medica.

[23] Wambersie A. (1999). ICRU report 62, prescribing, recording and reporting photon beam therapy (supplement to ICRU Report 50). ICRU News.

[24] Anderton J., Moroz V., Marec-Bérard P., Gaspar N., Laurence V., Martín-Broto J., Sastre A., Gelderblom H., Owens C., Kaiser S. (2020). International randomised controlled trial for the treatment of newly diagnosed EWING sarcoma family of tumours–EURO EWING 2012 Protocol. Trials.

[25] Behrends C., Haussmann J., Kramer P.H., Langendijk J.A., Gottschlag H., Geismar D., Budach W., Timmermann B. (2021). Model-based comparison of organ at risk protection between VMAT and robustly optimised IMPT plans. Z. Für Med. Phys..

[26] Acharya S., Wang C., Quesada S., Gargone M.A., Ates O., Uh J., Krasin M.J., Merchant T.E., ho Hua C. (2021). Adaptive Proton Therapy for Pediatric Patients: Improving the Quality of the Delivered Plan With On-Treatment MRI. Int. J. Radiat. Oncol. Biol. Phys..

[27] Heukelom J., Fuller C.D. (2019). Head and Neck Cancer Adaptive Radiation Therapy (ART): Conceptual Considerations for the Informed Clinician. Semin. Radiat. Oncol..

[28] Weistrand O., Svensson S. (2015). The ANACONDA algorithm for deformable image registration in radiotherapy. Med. Phys..

[29] Ger R.B., Yang J., Ding Y., Jacobsen M.C., Fuller C.D., Howell R.M., Li H., Jason Stafford R., Zhou S., Court L.E. (2017). Accuracy of deformable image registration on magnetic resonance images in digital and physical phantoms. Med. Phys..

[30] Brock K.K., Mutic S., McNutt T.R., Li H., Kessler M.L. (2017). Use of image registration and fusion algorithms and techniques in radiotherapy: Report of the AAPM Radiation Therapy Committee Task Group No. 132. Med. Phys..

[31] Bolan P. (2020). 3D Shepp–Logan Phantom. https://www.mathworks.com/matlabcentral/fileexchange/9416-3d-shepp-logan-phantom.

[32] Pearce M.S., Salotti J.A., Little M.P., McHugh K., Lee C., Kim K.P., Howe N.L., Ronckers C.M., Rajaraman P., Craft A.W. (2012). Radiation exposure from CT scans in childhood and subsequent risk of leukaemia and brain tumours: A retrospective cohort study. Lancet.

[33] Korevaar E.W., Habraken S.J., Scandurra D., Kierkels R.G., Unipan M., Eenink M.G., Steenbakkers R.J., Peeters S.G., Zindler J.D., Hoogeman M. (2019). Practical robustness evaluation in radiotherapy – A photon and proton-proof alternative to PTV-based plan evaluation. Radiother. Oncol..

[34] Simone C.B., Ly D., Dan T.D., Ondos J., Ning H., Belard A., O’Connell J., Miller R.W., Simone N.L. (2011). Comparison of intensity-modulated radiotherapy, adaptive radiotherapy, proton radiotherapy, and adaptive proton radiotherapy for treatment of locally advanced head and neck cancer. Radiother. Oncol..

[35] Volpe S., Bondiau P.Y., Claude L., Claren A., Padovani L., AlGhamdi H., Duhil De Benaze G., Opitz L., Baudin G., Dejean C. (2021). Postsurgical geometrical variations of tumor bed and brainstem during photon and proton therapy for pediatric tumors of the posterior fossa: Dosimetric impact and predictive factors. Strahlenther. Und Onkol..

[36] Hague C., Aznar M., Dong L., Fotouhi-Ghiam A., Lee L.W., Li T., Lin A., Lowe M., Lukens J.N., McPartlin A. (2020). Inter-fraction robustness of intensity-modulated proton therapy in the post-operative treatment of oropharyngeal and oral cavity squamous cell carcinomas. Br. J. Radiol..

[37] Vidal M., Moignier C., Patriarca A., Sotiropoulos M., Schneider T., De Marzi L. (2021). Future technological developments in proton therapy—A predicted technological breakthrough. Cancer/Radiothérapie.

[38] Price J., Hall E., West C., Thomson D. (2020). TORPEdO—A Phase III Trial of Intensity-modulated Proton Beam Therapy Versus Intensity-modulated Radiotherapy for Multi-toxicity Reduction in Oropharyngeal Cancer. Clin. Oncol..

[39] Aly F., Miller A.A., Jameson M.G., Metcalfe P.E. (2019). A prospective study of weekly intensity modulated radiation therapy plan adaptation for head and neck cancer: Improved target coverage and organ at risk sparing. Australas. Phys. Eng. Sci. Med..

[40] Rathod H., Mehta M., Kichloo A., Mankada S., Shah R. (2020). Adaptive Radiotherapy in Orbital Rhabdomyosarcoma: A Case Report. J. Med. Sci. Clin. Res..

[41] Shusharina N., Chan A., Adams J., Chen G., Sharp G. (2012). Adaptive Proton Radiation Therapy for Base of Skull Tumors. Int. J. Radiat. Oncol. Biol. Phys..

[42] Lamiman K., Wong K.K., Tamrazi B., Nosrati J.D., Olch A., Chang E.L., Kiehna E.N. (2016). A quantitative analysis of craniopharyngioma cyst expansion during and after radiation therapy and surgical implications. Neurosurg. Focus FOC.

[43] Ajithkumar T., Mazhari A.L., Stickan-Verfürth M., Kramer P.H., Fuentes C.S., Lambert J., Thomas H., Müller H., Fleischhack G., Timmermann B. (2018). Proton Therapy for Craniopharyngioma—An Early Report from a Single European Centre. Clin. Oncol..

[44] Elter A., Dorsch S., Mann P., Runz A., Johnen W., Spindeldreier C.K., Klüter S., Karger C.P. (2019). End-to-end test of an online adaptive treatment procedure in MR-guided radiotherapy using a phantom with anthropomorphic structures. Phys. Med. Biol..

[45] Meschini G., Vai A., Paganelli C., Molinelli S., Fontana G., Pella A., Preda L., Vitolo V., Valvo F., Ciocca M. (2020). Virtual 4DCT from 4DMRI for the management of respiratory motion in carbon ion therapy of abdominal tumors. Med. Phys..

[46] Wissmann L., Santelli C., Segars W.P., Kozerke S. (2014). MRXCAT: Realistic numerical phantoms for cardiovascular magnetic resonance. J. Cardiovasc. Magn. Reson..

[47] Putz F., Mengling V., Perrin R., Masitho S., Weissmann T., Rösch J., Bäuerle T., Janka R., Cavallaro A., Uder M. (2020). Magnetic resonance imaging for brain stereotactic radiotherapy. Strahlenther. Und Onkol..

[48] Mengling V., Bert C., Perrin R., Masitho S., Weissmann T., Mansoorian S., Siavooshhaghighi H., Janka R., Doussin S., Habatsch M. (2021). Implementation of a dedicated 1.5 T MR scanner for radiotherapy treatment planning featuring a novel high-channel coil setup for brain imaging in treatment position. Strahlenther. Und Onkol..

[49] Stanescu T., Hans-Sonke J., Stavrev P., Fallone B.G. (2006). 3T MR-based treatment planning for radiotherapy of brain lesions. Radiol. Oncol..

[50] Slagowski J., Ding Y., Wen Z., Fuller C., Chung C., Kadbi M., Ibbott G., Wang J. (2018). Quantification of Geometric Distortion in Magnetic Resonance Imaging for Radiation Therapy Treatment Planning. Int. J. Radiat. Oncol. Biol. Phys..

[51] Pappas E.P., Alshanqity M., Moutsatsos A., Lababidi H., Alsafi K., Georgiou K., Karaiskos P., Georgiou E. (2017). MRI-Related Geometric Distortions in Stereotactic Radiotherapy Treatment Planning: Evaluation and Dosimetric Impact. Technol. Cancer Res. Treat..

[52] Ulin K., Urie M.M., Cherlow J.M. (2010). Results of a Multi-Institutional Benchmark Test for Cranial CT/MR Image Registration. Int. J. Radiat. Oncol. Biol. Phys..

[53] Price R.G., Knight R.A., Hwang K.P., Bayram E., Nejad-Davarani S.P., Glide-Hurst C.K. (2017). Optimization of a novel large field of view distortion phantom for MR-only treatment planning. J. Appl. Clin. Med. Phys..

[54] Handrack J., Bangert M., Möhler C., Bostel T., Greilich S. (2020). Towards a generalised development of synthetic CT images and assessment of their dosimetric accuracy. Acta Oncol..

[55] Koivula L., Wee L., Korhonen J. (2016). Feasibility of MRI-only treatment planning for proton therapy in brain and prostate cancers: Dose calculation accuracy in substitute CT images. Med. Phys..

[56] Boye D., Lomax T., Knopf A. (2013). Mapping motion from 4D-MRI to 3D-CT for use in 4D dose calculations: A technical feasibility study. Med. Phys..

[57] Dolde K., Naumann P., Dávid C., Gnirs R., Kachelrieß M., Lomax A.J., Saito N., Weber D.C., Pfaffenberger A., Zhang Y. (2018). 4D dose calculation for pencil beam scanning proton therapy of pancreatic cancer using repeated 4DMRI datasets. Phys. Med. Biol..

[58] Neppl S., Landry G., Kurz C., Hansen D.C., Hoyle B., Stöcklein S., Seidensticker M., Weller J., Belka C., Parodi K. (2019). Evaluation of proton and photon dose distributions recalculated on 2D and 3D Unet-generated pseudoCTs from T1-weighted MR head scans. Acta Oncol..

[59] Spadea M.F., Pileggi G., Zaffino P., Salome P., Catana C., Izquierdo-Garcia D., Amato F., Seco J. (2019). Deep Convolution Neural Network (DCNN) Multiplane Approach to Synthetic CT Generation From MR images—Application in Brain Proton Therapy. Int. J. Radiat. Oncol. Biol. Phys..

[60] Hoffmann A., Oborn B., Moteabbed M., Yan S., Bortfeld T., Knopf A., Fuchs H., Georg D., Seco J., Spadea M.F. (2020). MR-guided proton therapy: A review and a preview. Radiat. Oncol..

[61] Wang C., Uh J., Merchant T.E., Hua C.h., Acharya S. (2021). Facilitating MR-Guided Adaptive Proton Therapy in Children Using Deep Learning-Based Synthetic CT. Int. J. Part. Ther..

[62] Spadea M.F., Maspero M., Zaffino P., Seco J. (2021). Deep learning based synthetic-CT generation in radiotherapy and PET: A review. Med. Phys..

[63] Bäumer C., Bäcker C.M., Conti M., Fragoso Costa P., Herrmann K., Kazek S.L., Jentzen W., Panin V., Siegel S.B., Teimoorisichani M. (2021). Can a ToF-PET photon attenuation reconstruction test stopping power estimations in proton therapy? A phantom study. Phys. Med. Biol..

